# Wild European Apple (Malus sylvestris (L.) Mill.) Population Dynamics: Insight from Genetics and Ecology in the Rhine Valley. Priorities for a Future Conservation Programme

**DOI:** 10.1371/journal.pone.0096596

**Published:** 2014-05-14

**Authors:** Annik Schnitzler, Claire Arnold, Amandine Cornille, Olivier Bachmann, Christophe Schnitzler

**Affiliations:** 1 Laboratoire Interdisciplinaire des Environnements Continentaux (LIEC) - UMR 7360, CNRS, Université de Lorraine, Metz, France; 2 Unicentre, University of Lausanne, Lausanne, Switzerland; 3 UMR 8079, CNRS, Orsay, France; 4 UMR 8079, Paris Sud University, Orsay, France; 5 Laboratory of Evolutionary Botany, University of Neuchâtel, Neuchâtel, Switzerland; 6 Laboratory Soil and Vegetation, University of Neuchâtel, Neuchâtel, Switzerland; 7 Sport Science Faculty, University of Strasbourg, Strasbourg, France; University of New South Wales, Australia

## Abstract

The increasing fragmentation of forest habitats and the omnipresence of cultivars potentially threaten the genetic integrity of the European wild apple (*Malus sylvestris* (L.) Mill). However, the conservation status of this species remains unclear in Europe, other than in Belgium and the Czech Republic, where it has been declared an endangered species. The population density of *M. sylvestris* is higher in the forests of the upper Rhine Valley (France) than in most European forests, with an unbalanced age-structure, an overrepresentation of adults and a tendency to clump. We characterize here the ecology, age-structure and genetic diversity of wild apple populations in the Rhine Valley. We use these data to highlight links to the history of this species and to propose guidelines for future conservation strategies. In total, 255 individual wild apple trees from six forest stands (five floodplain forests and one forest growing in drier conditions) were analysed in the field, collected and genotyped on the basis of data for 15 microsatellite markers. Genetic analyses showed no escaped cultivars and few hybrids with the cultivated apple. Excluding the hybrids, the genetically “pure” populations displayed high levels of genetic diversity and a weak population structure. Age-structure and ecology studies of wild apple populations identified four categories that were not randomly distributed across the forests, reflecting the history of the Rhine forest over the last century. The Rhine wild apple populations, with their ecological strategies, high genetic diversity, and weak traces of crop-to-wild gene flow associated with the history of these floodplain forests, constitute candidate populations for inclusion in future conservation programmes for European wild apple.

## Introduction

The wild apple (*Malus sylvestris* (L.) Mill.), a small fruit tree of the Rosaceae family, is a light-demanding, pioneer species of European forests. It is found in a broad range of latitudes between 370 and 640 N, soil environmental conditions and forest habitats, from deciduous (oak, hornbeam and lime) to mixed (deciduous and conifer) [1], [2], [3], [4], [5], [6], [7], [8], [9]. Given its high light demands and low competitive abilities, wild apple is naturally scattered in gaps within the forest and at the edge of the forest, or in extremely dry or wet sites at which other plants are less competitive [10], [11]. Most individuals are less than 10 m tall [12].

Unlike most upland forests, floodplain hardwood forests along large rivers provide favourable conditions for the establishment and reproduction of the European wild apple. Floods strongly limit the expansion of the most competitive European trees, such as beech (*Fagus sylvatica* L.), providing other flood-tolerant, light-demanding hardwood species, such as oak (*Quercus robur* L.), ash (*Fraxinus excelsior* L.), white poplar *(Populus alba* L.) and elm (*Ulmus minor* Mill.) with an opportunity to spread [13], [14], [15]. These forests are characterized by a multi-storey vertical architecture, dominated by light-demanding woody species. This architecture is shaped both by regular flooding and by extensive forest management (coppicing with standards) [13], [16], [17]. The wild apple is regularly recorded in these forests, at densities of one to two individuals per hectare [18]. Wild European apple populations are very rare in pioneer alluvial forests composed largely of members of the Salicaceae [17].

Interest in wild apple has recently grown, because population sizes for this tree have fallen considerably, particularly over the last two decades. The increasing fragmentation of forest habitats and the omnipresence of the cultivated apple (*Malus domestica* Borkh.) in the landscape have made crop-to-wild introgression more likely [19], [20], [21], [22], [23]. These threats have triggered investigations of the genetic diversity and structure of *M. sylvestris* across Europe. Genetic analyses across the geographic distribution range of this species have revealed the presence of three main populations, displaying high levels of genetic diversity in Europe: one covering a large area of Western Europe and the other two covering limited areas in Eastern Europe, one in the Balkans and the other in the Carpathian Mountains [24]. However, investigations of the ecology and genetic diversity and structure of local populations are required to determine the most suitable conservation policies for this species at different scales. These questions have already been addressed in some European countries (*i.e.*, Belgium and the Czech Republic), which have classified *M. sylvestris* as an “endangered species”, leading to the dedication of greater resources to *in situ* and *ex situ* conservation programmes [11], [25].

Floodplain forests, which are dominated by light-demanding canopy trees, are candidate areas for the targets of European wild apple conservation programs. In the Upper Rhine Valley (France), the European wild apple grows preferentially in these floodplain forests, along the Rhine and its tributaries [18], and in the oak forests of the alluvial terraces created during the last ice age (*i.e.*, the Würm period) [26]. Deep within the forest, all mature individuals flower in spring, but fruiting is less frequent than at the edge of the forest [18], [26]. This species generally displays a strongly clumped distribution (personal communication, Felix Bersier). Populations typically have an unbalanced age structure, most of the trees being old (>100 years), with only very rare seedlings and saplings. We hypothesize that the current regeneration problems for this species may be linked to three main factors: i) changes in genetic diversity due to introgression, ii) river management, and iii) changes in forestry practices.

We investigated the ecology, age structure and genetic diversity of *Malus sylvestris* in the forests of the Upper Rhine Valley, France. These forests are potential candidate areas for inclusion in future wild apple conservation programmes. With this in mind, our main objectives were: (1) to evaluate the chances of the current populations surviving and (2) to determine the genetic characteristics of the wild populations of the Rhine Valley, by checking for the presence of hybrids or feral individuals and evaluating the genetic diversity of the wild apple. On the basis of the results obtained, we aim to reconstruct the history of this species over the last century and to provide information useful for future conservation actions in this region.

## Materials

### The Study Area

**Figure 1:**
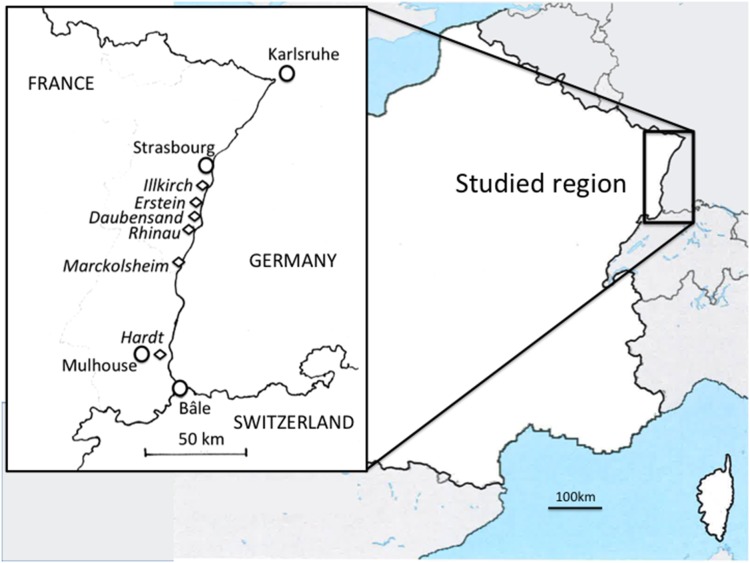
Map of the sampling populations in the Rhine Valley, France.

The study site is located in the Upper Rhine Rift Valley, in a rift, on the French side of the river (Figure 1). Trees were sampled in six nutrient-rich deciduous forests distributed along a 100 km North-South transect (latitude N 48°05′−47°83′; longitude E 7°30′−7°43′, respectively) along the Rhine river. The altitude increases from North to South from 144 m to 236 m (Table 1). The southernmost site is a large Rhine terrace created during the last glacial period (the Hardt forest). About 50 km from the Hardt, five big forests stretch along the Rhine in the Holocene floodplain (the Marckolsheim, Rhinau, Daubensand, Erstein and Illkirch forests).

**Table 1:** Altitude, geographical locations and surfaces of the six study populations in the RhineValley.

Sites	altitude (m)	lat. N; long. E	Forest area (ha)	Surface area (ha)
Illkirch	145 m	48.52; 7.75	811	500
Erstein	152 m	48.43; 7.73	360	180
Daubensand	160 m	48.34; 7.73	427	100
Rhinau	160 m	48.25; 7.67	250	50
Marckolsheim	175 m	48.15; 7.57	690	100
Hardt	232 m	48.05; 7.30–7.43	13040	500

The mean altitude of the Hardt forest terrace is 230 m. The climate is more continental than at the sites further north, with a mean annual rainfall of only 600 mm and 1800 hours of sunshine per year [27]. Incision processes caused a significant (15 m) lowering of the water table within glacial coarse sediments during the Holocene, accounting for the lack of availability of underground water for trees. Plants in this area must cope with general dryness during the vegetative season, and this has resulted in a total absence of beech trees, for example. Forests of the area have been defined through the European phytosociological context. Phytosociology is the branch of science which deals with the classification of plant communities, their composition and development. The basic unit of syntaxonomy is the “association”. The association is a conceptual model of a concrete phytocoenosis (the plant component of a biocoenosis). Associations with floristic and territorial affinities can be grouped in larger ecological conceptual units (*i.e.*, syntaxa) called “alliances”. Similar alliances may be grouped in “orders” and orders in vegetation “classes” [28]. In the Hardt, the major alliance is the Carpinion, dominated by oaks (*Quercus petraea* (Matt.) Liebl*; Q. pubescens* Willd) and hornbeam (*Carpinus betulus* L.). The understorey is relatively light and rich in Rosaceae species (*Malus sylvestris, Pyrus pyraster L., Crataegus monogyna* Jacq, *Sorbus torminalis* Crantz and *Prunus spinosa* L.).

The mean altitude of the Holocene floodplain is 148 m. The climate is temperate and oceanic, with a mean annual temperature of 11°C and a mean annual rainfall of 730 mm. The number of hours of sunshine varies between 1600 and 1700 per year [27]. The hydrological regime of the Rhine is nival and nivo-glacial, with summer flooding. Substrates are calcareous and coarse-textured. Two phases of river management have occurred. In the 19^th^ century, straightening and embankment works modified the hydrological and sedimentary processes considerably, with a general deepening of groundwater levels. The main consequence of these changes was an increase in the surface area covered by mature hardwoods (Alno-Ulmion alliance), which had previously been restricted to high terraces close to the river. The second phase occurred in the middle of the 20^th^ century (1940–1960), with the canalisation of the river. This totally eliminated flooding, except on small islands. This elimination of flooding and stabilisation of ground water fluctuations resulted in the hardwood canopy becoming more uniform, with a large increase in the density of sapling carpets, which were previously selected by temporal anoxia during floods. Early successional forests, dominated by the Salicaceae (*i.e.*, the Salicion alliance), are now restricted to a few old channels [14], [29].

The forests of the Rhine Valley were coppiced until the mid 20^th^ century. A period of intensive management (*i.e.*, oak/ash/maple plantations with large areas of clear-cutting) then followed from the 1970s to the 1980s and 1990s, particularly in certain forests, such as that at the Daubensand site. Management practices have since shifted towards other forestry practices, and this has resulted in a forest of heterogeneous age, with small clear-cuts, natural regeneration and woody vines. Natural reserves have been created in the Rhine forests, mostly bordered by dikes, in which the complex architecture of hardwoods remains visible. Wild boar and roe deer populations are abundant in the Rhine Valley, facilitating the active dispersal of wild apple fruits from late August to late September. We chose to study these protected areas here.

On both sides of the river, over a distance of 180 km, 45530 ha of the Upper Rhine Valley have been classified as a Ramsar site. The Rhine forests (both the floodplain and the Hardt forest) have also been integrated into the Natura 2000 conservation network in France.

There were many orchards in the Rhine Valley before the 1970s, covering at least one fifth of the total area of cultivated land. Cultivated apple trees were also planted with other fruit trees, as landmarks, and along forest edges. They were traditionally used for fruit consumption and distilling apple brandy, as well as for decoration. These different uses account for the large number of cultivars grown. Since the 1970s, some of these orchards have been replaced by maize crops.

### Malus Sylvestris

This species has a long juvenile period, lasting five to six years. The flowers are hermaphrodite and pollinated predominantly by bees and flies (Syrphidae) [30]. This is an outcrossing species, with a gametophytic self-incompatibility system preventing self-fertilisation [31], [32], [33]. Dispersal is strictly endozoochorous. The animals dispersing the seeds are large mammals, such as ungulates, brown bears and humans [20], [30].

In the Rhine forests of the Holocene plain and the Hardt, this species reproduces every year. Apple production is nevertheless highly variable, depending on the meteorological events of the previous spring and summer temperatures [26]. In suitable climatic conditions, such as those occurring in 2002 and 2003, almost all the individuals flowered and produced large numbers of fruits in a synchronous manner. In cold springs and summers, far fewer apples are produced and fruit production is also limited to a few individuals. Ungulates feed on apples, but only in small quantities, probably because of their acidity. As a result, many apples rot beneath the parent plants, with no germination of the seeds [18].

**Figure 2:**
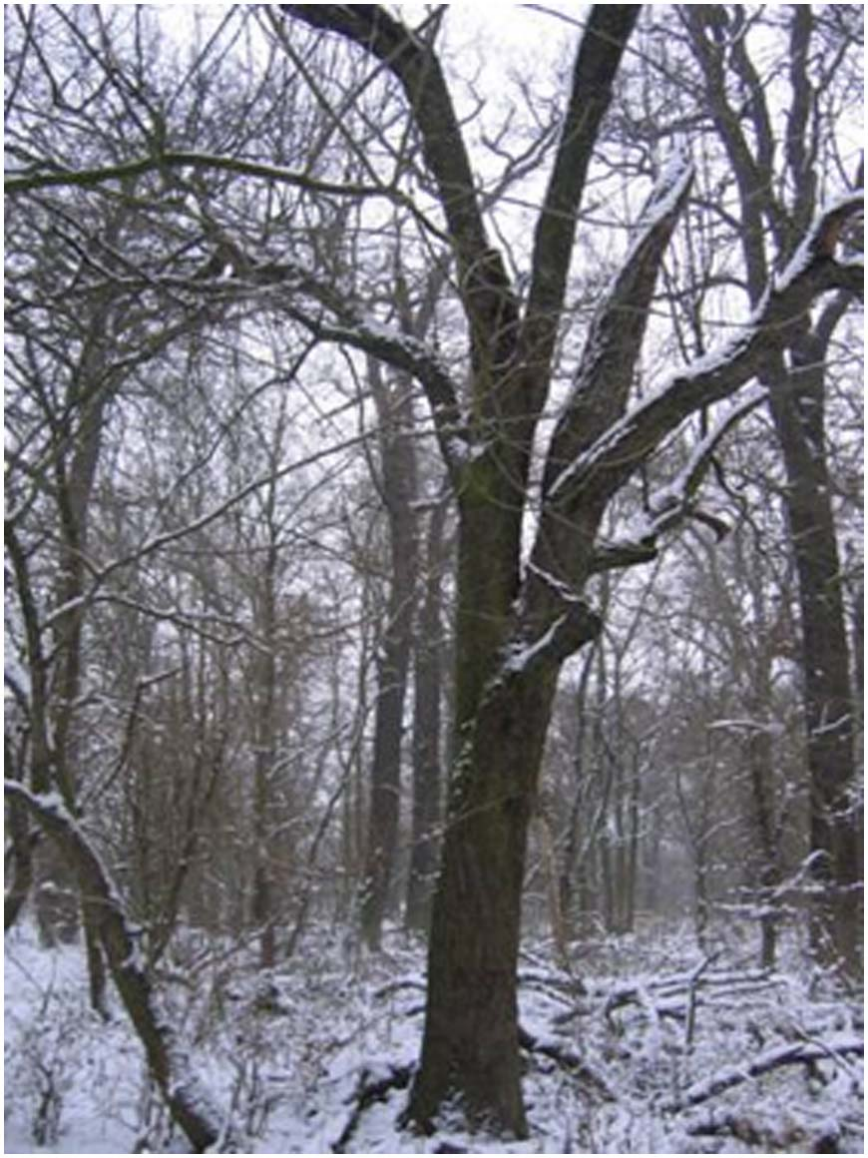
Healthy wild apple tree with a straight, upright trunk, Erstein forest (photo Annik Schnitzler).

**Figure 3:**
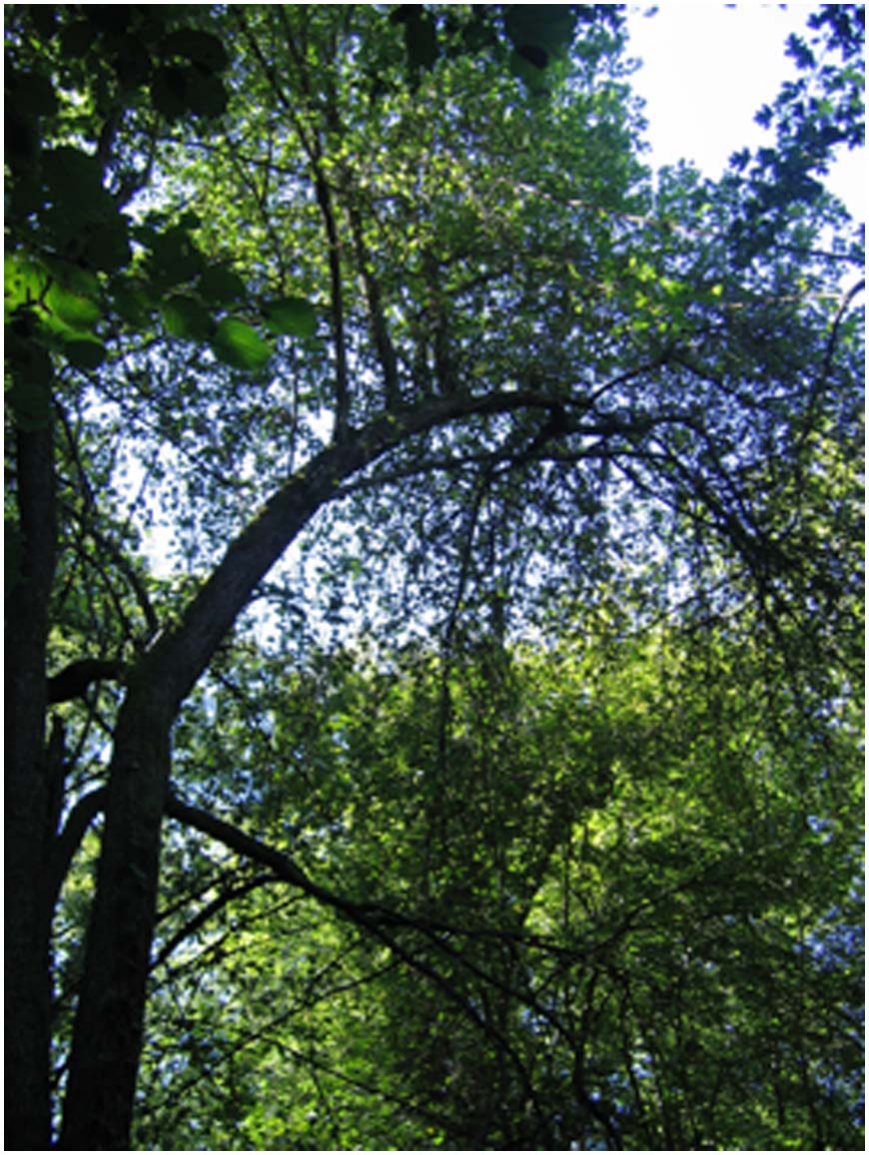
Wild apple tree with a reiterated trunk, Erstein forest (photo Annik Schnitzler).

Adult trees present various sizes and morphological forms. They may have upright trunks (Figure 2) and some very large individuals have been known to reach heights of up to 21 m. However, many individuals develop inclined trunks with a reiterated (as defined in [34]) vertical axis (Figure 3). These two morphological forms can be interpreted as a plastic response to light availability. Trunks are straight when suitable light conditions prevailed during growth and maturity and individuals with this morphological form are generally in good health. Inclined trunks (generally leaning towards an adjacent source of light) are found in situations of overtopping. Individuals with inclined trunks are more vulnerable to breakage, resulting in attacks by pathogenic bacteria (*Pseudomonas* sp.) or fungi and, ultimately, mistletoe (*Viscum album* L.). These individuals are therefore considered to be in bad health. Wild apples are often colonized by large woody vines (*Hedera helix* L. and/or *Clematis vitalba* L.), which climb up to the crown. These vines need good light conditions to ascend the trunks in understoreys. Their presence, together with that of large apple trees, thus provides an indirect evaluation of the suitability of the local light microclimate, mostly in gaps or ecotones. When ivy, which is more shade-tolerant than other climbers, is present alone, the under storey is often illuminated from the side [35].

Dendrochronological studies in the Erstein natural reserve (unpublished report 2007, Patrick Gassmann) (Figure S1) have shown wild apple trees grow quite slowly during the first 30 years of their life, subsequently growing more rapidly between the ages of 30 and 50 years. Beyond this age, their growth rates decrease significantly, but this could possibly be due to the changes in the hydrological regime caused by the canalisation of the Rhine, as observed for oaks [36]. Senescence seems to occur after 100 years. Mean increase in diameter, between the ages of 90 and 150 years, has been estimated at only 1.21 mm per year. According to Gassmann’s unpublished data (2007), individuals with a diameter at breast height (DBH) of more than 55 cm and with heights of 15 to 20 m in the floodplain forest are all at least 100 years old, with the age of the oldest tree estimated at 156 years.

## Methods

### Data Collection

We have obtained permission for collecting wild apple leaves from the Conservatoire des Sites Alsaciens, which is the authority responsible of the natural reserves of Erstein and Rhinau, manager of the Erstein natural reserve and the Rhinau natural reserves.

The other locations did not require specific permission because forests are not protected but we have collected the leaves with the help and consent of local foresters from the Office National des Forêts, manager of these forests. These field studies did not involve endangered or protected species.

We collected data for 255 trees growing at six different sites in the forests of the Rhine Valley: the Hardt (*N* = 80), Marckolsheim (*N* = 47), Rhinau (*N* = 3), Daubensand (*N* = 3), Erstein (*N* = 80) and Illkirch (*N* = 42) forests. The small number of individuals from the Rhinau and Daubensand forests reflects the small number of wild apple trees present at these sites. This scarcity of wild apple is due to highly hydromorphic conditions at Rhinau and local intensive forest management in Daubensand. The geographical coordinates (latitude and longitude) of each site and the DBH of each individual were recorded.

### Genetic Analysis

#### DNA extraction and microsatellite genotyping

Leaves were dried in the presence of silica gel. Genomic DNA was extracted with the Nucleo Spin Plant DNA Extraction Kit II (Macherey & Nagel, Düren, Germany), according to the manufacturer’s instructions. Microsatellites were amplified by multiplex PCR, as described by Cornille et al. [22]. A total of 255 individual wild apple trees and 15 microsatellites loci were retained for the statistical analysis.

#### Identification of hybrids

Gene flow from the cultivated apple *Malus domestica* to the European wild apple has been demonstrated [22]. We therefore initially carried out a STRUCTURE 2.3.3. analysis [37], including 40 reference *M. domestica* cultivars previously identified as displaying no introgression from the European wild apple (*i.e.*, with membership coefficients >0.9 to the *M. domestica* genepool) [22] and the entire *M. sylvestris* dataset (*N* = 255). We retained only individuals assigned with a membership coefficient of more than 0.7 to the *M. sylvestris* genepool for further genetic analyses (*N* = 246).

#### Analyses of the genetic diversity of wild apples

After removing the individuals identified as potential hybrids, we used GENEPOP 4.0 [38] to calculate heterozygosities (expected (*H_E_*) and observed (*H_O_*)), Weir & Cockerham *F*-statistics, deviation from Hardy-Weinberg equilibrium and genotypic linkage disequilibrium. Allelic richness and private allele frequencies were calculated with ADZE software, for a sample size of 4 (2×2 chromosomes) [39]. In parallel, we performed frequency-based assignment (Paetkau test) with GenAlEx 6.5 [40], to assign individuals to the population to which they had the highest likelihood of belonging.

#### Analysis of population structure

We used the individual-based Bayesian clustering methods implemented in STRUCTURE 2.3.3 [37] to investigate intraspecific population structure and admixture. This method is based on the use of Markov Chain Monte Carlo (MCMC) simulations to infer the assignment of genotypes to *K* distinct clusters. The underlying algorithms attempt to minimise deviations from Hardy-Weinberg and linkage disequilibria within each cluster. We used an admixture model with *priors*. Six independent analyses were carried out for each number of clusters *K* (1≤*K*≤6), with 100000 MCMC iterations after a burn-in of 100000 steps.

### Ecological Analysis

#### Size variation between populations

The significance of differences in DBH between the floodplain forests (*N* = 175 trees) and the Würmian terrace (*N* = 80) was assessed with Student’s *t*-tests.

#### Collection of ecological data from the erstein site

Ecological data were collected at a finer scale, in the natural reserve of Erstein (180 ha). Almost all the individuals present in this forest (227 individuals) were studied. We investigated possible associations between size, health status, morphological forms and the presence of woody vines, by collecting the following data: DBH, health status (a tree was considered as (i) in good health if upright and devoid of fungi, mistletoe, or/and dead axes; (2) in bad health when leant and reiterated axes. These trees were attacked by fungi or mistletoe and often harbour holes and dead axes), morphological plasticity, presence of *Hedera helix* and/or *Clematis vitalba*, and forest architecture above the tree (gap *vs* overtopping canopy). We then performed a hierarchical clustering analysis (HCA). The squared Euclidean distance dissimilarity measure and the Ward linkage method were applied to determine profiles. We then checked for uniform variance and normality of the distribution and carried out a series of one-way ANOVAs to investigate the differences between categories for the variables studied. The significance level was set at *P*<0.05. We identified four categories, which we used in subsequent analyses.

Spatial correlation was assessed on the basis of geographical data, using the four categories identified, in a Mantel test. This test assessed the correlation between a similarity matrix containing the four categories of wild apples and the converted geographical distance matrix (Si = 1-Di).

## Results

### Genetics

#### Small number of hybrids

STRUCTURE analysis detected nine hybrids (5%, *i.e.*, individuals assigned with a membership coefficient of more than 0.3 to the *M. domestica* genepool) among the entire sample of trees from the population of the Rhine Valley. The nine hybrid individuals were located in the Erstein (1), Hardt (7) and Illkirch (1) forests. These hybrids were not included in subsequent genetic analyses.

All the hybrids from the floodplain were healthy and had a relatively large DBH (from 55 to 124 cm), indicating an age of almost or more than 100 years. With the limited number of loci studied, it was difficult to determine in which generation introgression had occurred, but our findings clearly indicate that the hybrids were fertile and that their presence could therefore lead to the presence of wild introgressed individuals in forests. In the Hardt, all the hybrids were juveniles aged between two and five years. Five of these hybrids were located along paths running within the forest, whereas two others were located within the interior of the forest.

#### High levels of genetic diversity and weak population structure in european wild apples

High levels of genetic diversity were observed at the six populations, with a global estimation of 0.77 (min: 0.74; max: 0.90). Heterozygosity was higher in the southern populations (Hardt), decreasing towards the north (Table 2). Frequency-based assignment revealed that the individuals of the Hardt forest were assigned to their own population with a frequency of 70% (Paetkau test; Table 2, ASS Self Pop). In Marckolsheim (53%), Erstein (37%), Illkirch (38%), Rhinau (0%) and Daubensand (0%), this proportion was lower or zero. Allelic richness did not differ between populations (Wilcoxon sign-rank tests, *P*<0.001). *F_IS_* was low, suggesting random mating within each population and a weak population structure (Table 2).

**Table 2:** Summary of genetic variation within each population.

Sites	N	ASS SelfPop	H_E_	H_O_	F_IS_	Ar
Hardt	73	70%	0.79	0.78	−0.016	2.83
Marckolsheim	47	53%	0.77	0.76	−0.017	2.80
Rhinau	3	0%	0.90	0.81	0	2.92
Daubensand	3	0%	0.78	0.78	0	2.88
Erstein	79	38%	0.77	0.78	0.008	2.84
Illkirch	41	37%	0.74	0.77	0.038*	2.85
Total	246		0.77	0.78	0.0048	

N: Number of individuals per populations, ASS Self Pop: percentage of individuals assigned to its own population (Paetkau test), *H_O_* and *H_E_*: observed and expected heterozygosities, respectively, *F_IS_*: inbreeding coefficient, *A_r_*: allelic richness averaged across loci, estimated by rarefaction using a standardized sample size of 4, *: *P*-value<0.001.

**Figure 4:**
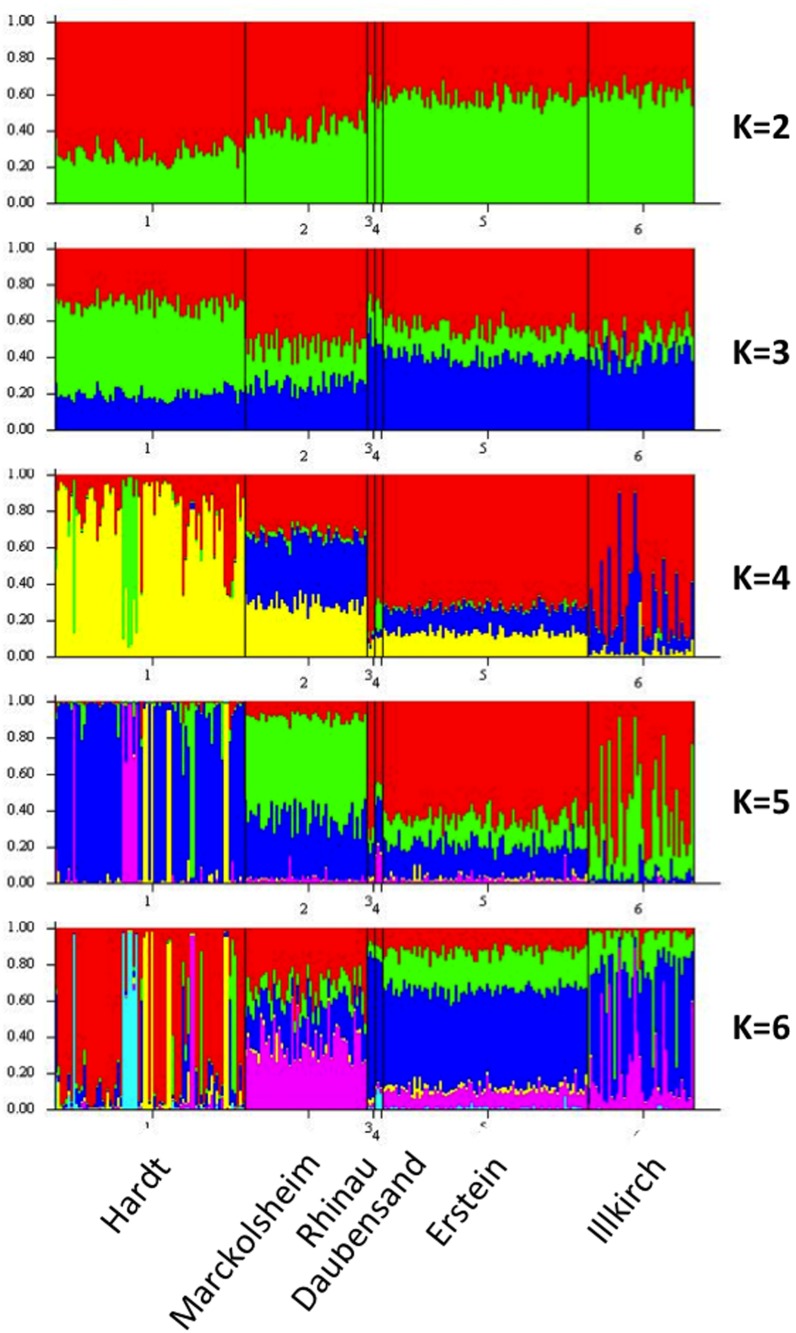
Population structure of *Malus sylvestris* (*N* = 246, 6 populations across the Rhine Valley), inferred with the Bayesian clustering algorithm implemented in STRUCTURE. Each individual is represented by a vertical bar, partitioned into *K* segments representing the proportions of ancestry of its genome corresponding to *K* clusters.

In STRUCTURE analysis with *priors*, the individuals of the floodplain populations (Marckolsheim, Rhinau, Daubensand, Erstein, Illkirch) were evenly distributed between the different clusters (visualised by different colours in Figure 4). Most individuals of the Hardt population were fully attributed to one particular cluster.

Given the small numbers of individuals in the Rhinau and Daubensand populations, caution is required in the interpretation of results for these populations.

### Morphological Traits and Distribution

**Figure 5:**
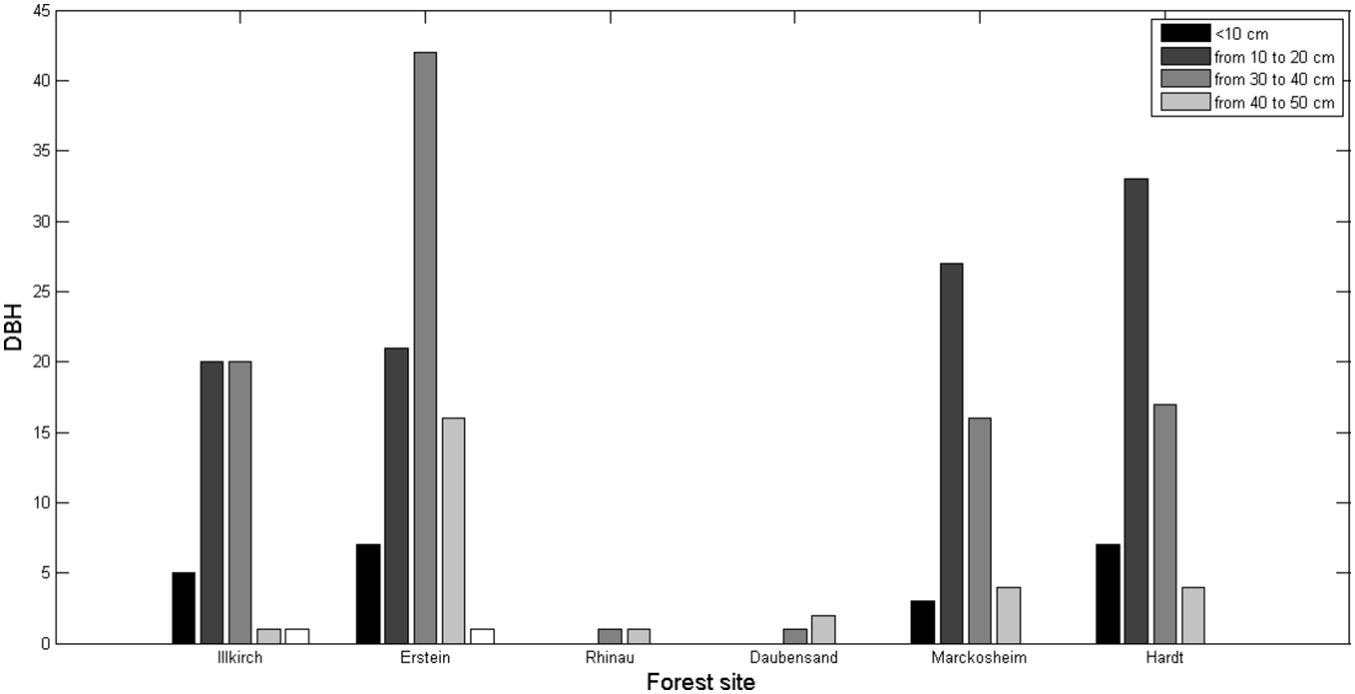
DBH distribution for the six populations of the Rhine valley, France.

We measured 255 individuals at the six populations: about 35% of the trees had a DBH between 10 to 20 cm, and an additional 35% had a DBH between 20 and 30 cm. Larger trees (DBH of 44 to 62 cm) were much rarer (only two individuals). Juveniles (*i.e.*, DBH<10 cm) were geographically dispersed, and saplings (from 50 cm to 1 m high) were found only in the Hardt forest. The tallest trees (21 m high, with a DBH of 27 cm) and the trees with the greatest DBH (44 cm, for a height of 17 m) were found in the natural reserve of Erstein (Figure 5). We separated the trees into two groups for analysis (floodplain *vs* Würmian terrace) and found a significant difference in DBH between these two groups, with higher values obtained for trees from the floodplain forests (mean ± standard deviation: 23.5±8.3) than for those from the Hardt forest (18.5±6.8) (*Student’s t*  =  *P*<0.05).

Cluster analysis for the Erstein forest revealed the presence of four distinct ecological categories of wild apples (Table 3). Category 1 (17%) comprised healthy individuals living in optimal light conditions, generally in gaps in the forest. The trees were large, with a DBH>30 cm. The trunks were straight and tall, with no reiteration. Both *Hedera helix* and *Clematis vitalba* had colonised the crowns of the wild apple trees of this category. Category 2 (33%) differed from Category 1 for one factor: the absence (or the very small dimensions) of the most light-demanding woody vine, *Clematis vitalba*. Category 3 (38%) contained stressed, parasitized trees, with multiple reiterations. Trees of all diameters (and ages) were observed. Ascending vines were rare. Category 4 (14%) was small, and contained healthy trees with upright trunks but without vines.

**Table 3:** Ecological characteristics of the four categories of wild apple trees in the Erstein forest.

Ecological characteristics	C1	C2	C3	C4
DBH (cm)	>30	>30	from 10 to 45	from 10 to 20
Healthstatus	good	good	bad	good
Morphological plasticity	Erect trunk	Erect trunk	reiterated axis	Erect trunk
Presence of *Hedera helix*	yes	yes	rare	No
Presence of *Clematis vitalba*	yes	no	no	No
Presence of *Viscum album*	no	no	yes	No
Forest architecture above the tree	large gap	small gap	overtopping	small gap

The Mantel *r* statistic (−0.0779) and the corresponding two-tailed *P* values (0.001) obtained for the 10000 permutations indicated that the four categories, taken together, were not randomly distributed in the forest. We tested the four categories separately against geographical distance: Category 4 was randomly distributed, whereas Categories 1, 2 and 3 were geographically constrained.

## Discussion

### Wild Apple Population Dynamics: An Original Ecological Strategy

The current ecological clustering pattern at the wild apple sites of the Erstein forest can be interpreted in terms of specific local history and environmental factors:

C1. This category established in large gaps. Before river management, such gaps were numerous because floods accelerate tree fall. After river management, the massive death of canopy elms following the spread of Dutch elm disease in the 1970s and 1980s also created large gaps. These large gaps were highly suitable for the establishment of both wild apples and light-demanding woody vines, such as *Clematis vitalba* in particular. These *Clematis vitalba* vines have now reached heights of 40 m and widths of more than several tens of meters, with trunks of more than 15 cm in diameter. *Hedera helix* was also favoured by the light conditions and by the cracked bark of the largest wild apple tree trunks. The extensive network of climbers growing in the canopy has prevented canopy closure for decades, limiting the overtopping of wild apples.
C2. The absence of *Clematis vitalba* suggests poorer light conditions, due to smaller gaps (or small clear-cuts created by extensive forestry practices), partial canopy closure or the presence of edges deep in the forest (between channels and the forest and between paths and the forest). However, there is nevertheless sufficient light for wild apple trees to grow straight up and for ivy to establish itself in the crown. This situation was found to be more frequent in the forest along the ancient river channels of the Rhine, in the eastern part of the reserve. In managed forests, human practices have essentially reproduced the heterogeneity of the canopy topography (through coppicing and the creation of small gaps), and respect for native trees has also preserved many wild apple sites.
C3. The wild apple trees of this category are in poor health because of poor local light conditions (for example, because of prolonged overtopping) and other unknown reasons (abrupt local changes in groundwater depth, for example). *Hedera helix* may or may not be present. Such situations were found in the best preserved part of the forest, rich in large, old canopy trees.
C4. This category results more from the age structure of wild apple populations in this region rather than a given ecological situation. All the individuals of this category are younger than those in the previous categories. They may have colonized sites with partially closed canopies or small temporary gaps or clear-cuts. These growth conditions allow the vertical growth of the trunk and are found at random sites in many parts of the forest. The absence of ivy is not an indication of low light conditions, but of the smoothness of the bark, making it difficult for the adventitious roots to establish a hold.

These ecological categories provide new insight into the factors driving the population dynamics of the European wild apple in the Rhine Valley:

1. *Wild apple ecology*. Beyond the local presence of specific communities of species associated with the different categories found (*i.e.*, C1 to C4), the differences in size between the floodplain and the Hardt populations may provide insight into the types of environment most suitable for wild apple in terms of water and nutrient resources. The moist and nutrient-rich soils of the floodplains seem to be highly suitable for rapid plant growth, contrasting markedly with the drier Hardt environment. Nevertheless, further ecophysiological investigations are required to determine the ecological preferences of wild apples.
2. *Local site-specific (anthropogenic or natural) historical events*. There were probably a large number of suitable sites for wild apple 150 years ago because 1) regular flooding severely limited woody plant regeneration [13] and thus competition and 2) the forest was managed by coppicing, increasing the likelihood of wild apple germinating and becoming established.

Successive floods in past decades may explain the unbalanced age structure of the populations. Following canalisation of the river, competition between seedlings increased, due to both the elimination of flooding and the spread of Dutch elm disease, which killed many elms, thereby creating large gaps in the canopy. In the meantime, forestry practices shifted from small to large clear-cuttings and plantations. These changes had deleterious effects on wild apple populations, decreasing sapling establishment and adult growth, and increasing stress in adults due to overtopping, except in large gaps created by the death of elms or extensive clear-cutting. The ecological conditions of the Erstein forest have thus resulted in a rarity of seedlings and saplings. A few 1 m- to 3 m-tall saplings were found in the driest parts of the forest, along paths. A few seedlings were also found in the dense carpets of saplings of other species, but they were heavily browsed and rapidly disappeared [18]. This suggests that the survival conditions for young individuals were degraded by the elimination of flooding, resulting in a significant decrease in natural gap dynamics and an increase in competition with other species at the juvenile stage. The death of elms favoured wild apple growth locally for a few years, but the canopy then rapidly closed, due in part to changes in forestry practices (small clear-cuttings) or strict protection. This situation may also reflect the natural strategy of this species (rarity of reproduction and germination and high vulnerability of saplings, compensated by the synchronous production of large amounts of apples in favourable years and the great age of adults).

Local high-disturbance events that temporarily open the canopy, such as high winds, fire and human practices, have also helped to maintain the light-demanding wild apple in these forests. More open, anthropogenic landscapes provide this species with suitable sites for survival.

The wild apple has thus developed a particular colonising strategy in deciduous forests, due to its low growth capacity and poor shade tolerance (resulting in scarcity within the forest and a tendency to grow in clumps), leading to an unbalanced age structure with many very old adults. This strategy accounts for the broad distribution of this species in Europe and the large differences in population density as a function of the light regime of the understorey and natural and anthropogenic disturbance or stress regimes.

### Wild Apple Population Dynamics: The Contribution of Genetic Data

The high heterozygosities of the Rhine and Hardt forests are consistent with the results of previous studies wild apple populations in Belgium [20], Denmark [30] and throughout Europe [24]. These Rhine populations are also included in the previously described Western European meta-population displaying a high level of genetic diversity [31].

STRUCTURE analysis revealed an absence of genetic structure across the Rhine forest. These results are consistent with the obligatory cross-pollination system of apples, preventing local endogamy, and the strict endozoochory by mammals over large territories. Large-scale dispersal increases opportunities for finding suitable forests in which individuals can reproduce at higher rates and sapling mortality is lower. The weak population structure revealed that, despite the high level of clumping observed in all forests, individuals from the same clump were not more closely related genetically than individuals from different clumps.

The ecological data indicated that the Hardt populations had a very different history from the floodplain populations. Indeed, before the dikes were built, hardwoods were also regularly destroyed by major floods (occurring several times per century) [41], and replaced by early successional Salicaceae forests unsuitable for wild apple [17]. This ecological situation, typical of floodplain forests on large river plains, contrasts markedly with the forests of the Hardt, in which conditions have remained more stable and suitable for wild apple. The Hardt forest is also significantly richer in wild apples than other upland forests of the Rhine plain because of the absence of shade-tolerant beech [26], as this forest probably acted as a major source of material for recolonisation after each major flood.

Several factors may account for the rarity of introgressed wild apples in the forests of the Rhine Valley: the sample size used, which was restricted to a narrow geographical area, and the only partial overlap in flowering time (*i.e.*, 1 or 2 weeks) between cultivated and wild apples [18]. More suitable conditions existing only a few decades ago, such as more anthropogenic openings in the forests at the beginning of the 20^th^ century, may account for the old age of the few hybrids we detected. It is also possible that the domesticated apple cannot tolerate the ecological constraints of the Rhine forest, limiting its presence in this area. Cultivars originated from *Malus sieversii* (Ledeb.) M. Roem from the Tian Shan Mountains [23], [42], [43], [44]. *Malus sieversii* is a very demanding species, and the main component of fruit-tree forests of these regions. These forests, characterized by an open canopy, are found on the moist slopes and alluvial plains of the mountain rivers, at altitudes of between 800 and 1500 m above sea level, in humus-rich soils [45]. In optimal light conditions, trees reach heights of 12 to 14 m with a DBH of 70 to 80 cm [44]. The cultivated apple may have an ecological tolerance profile similar to that of its ancestral progenitor, preventing it from growing under the closed and dense canopy of European forests (even if its seeds are dispersed by ungulates) or escaping and becoming established in competition with native saplings. Thus, 4000 to 8000 years of domestication (possibly 80–150 generations) may not have been sufficient for the adaptation of feral domesticated individuals to conditions in the wild. The European wild apple was recently shown to be the second main contributor to the germplasm of cultivated apple [22], suggesting a potential source of introgression of adapted traits to local conditions in Europe, but this requires further investigation [44]. However, feral hybrids were very rare deep within the forest. These individuals may thus have become established along paths, where competition is weak, or due to local protection by foresters.

## Conclusion: The History of Wild Apples in the Rhine Valley

Our results suggest that the current difficulties encountered in wild apple regeneration in the Rhine forests do not result from hybridization. However, two other factors (hydrological changes and changes in forestry practices) have made a major contribution to these difficulties. We propose here a possible reconstruction of the principal events leading to the current situation as concerns wild apple in the Upper Rhine floodplain forests:

1. Historically, and even during prehistory, before human management of the river, the wild apples lived in hardwood forests towards the outer edges of the floodplain. These populations were regularly destroyed by major floods, but subsequently colonized the floodplain from source sites, such as the Hardt forest in particular, through dispersal by large mammals.
2. Once the dikes had been built, flood strength decreased, favouring the expansion of hardwoods. This period was ideal for the wild apple, particularly in the zones between two dikes, in which the hardwoods were still flooded, as it limited the penetration of shade-tolerant species and seedling survival, and forest management was dominated by a combination of coppicing and high trees. Most of the current population of wild apples probably dates from this period, with establishment and reproduction occurring from 1850 to 1960.
3. After canalisation, the opportunities for establishment decreased, and many saplings and adult trees died. However, the light regime in the understoreys remained adequate for the maintenance of an adult population in gaps, forest ecotones, clear-cuts created by intensive forestry and overtopping situations in closed forests.
4. Since the establishment of regulations protecting the ecosystem of the Rhine Valley in the 1990s, the forests have been managed less intensively, and wild apples are not cut anymore, with their survival even favoured locally by the cutting of overtopping trees. However, competition remains fierce in the sapling carpet, due to the absence of flooding. The age structure of the population thus remains unbalanced and many of the apples in the most productive parts of the plain may soon become senescent.
5. However, hybrids and cultivars are clearly disadvantaged in these forests, and so the genome of the wild apple is thus well preserved.
6. In the Hardt forest, ecological conditions for wild apples have changed little, other than an increase in fragmentation of the area due to paths, roads, meadows and cultivated fields, facilitating the penetration of hybrids. Hybrids may thus be more numerous in this forest than in the floodplain, and this higher frequency of hybrids may also reflect lower levels of interspecific competition than in the humid conditions of the floodplain.

The most effective way to preserve this species at the European scale would be to recognise this species as threatened and to protect all forest types naturally rich in wild apples against fragmentation. An essential condition is that these forests must be wide enough (*i.e.*, at least 100 ha) and preferably circular in shape, according to our observations in the Rhine floodplain [14], for the maintenance of full functionality, to which wild apples are adapted. Cultivars and hybrids are not competitive enough to become established in such an environment.

In floodplains, the re-creation of erosive zones in alluvial forests would improve the establishment of wild apple saplings by increasing gaps dynamics and decreasing sapling competition. In both upland and alluvial forests, a return to natural forest dynamics would be ideal, because this situation most closely resembles natural conditions. Alternatively, extensive forestry practices could be introduced.

## Supporting Information

Figure S1 **Diagram of dendrochronological sequences from wild apple trees in the Erstein forest (Kuhau site).** The nine dendrochronological sequences shown in this diagram illustrate the growth characteristics of wild apples in the floodplain forest of Erstein. The horizontal line, which represents a growth ring with a width of 1 mm, provides a benchmark for assessing periods of growth and no growth. The circle drawn to the left of each sequence indicates the estimated pith location, as coring to the pith could not be done because of the hardness of the wood. The first five to ten years of difficult growth are generally followed by 70 years of vigorous growth and then a final 40 years or so of slower growth, as evidenced by very thin rings (example of KUHAU-029). The great variations in growth for each of the analysed wild apples made it impossible to establish correlations among their dendrochronological sequences. These sequences were thus positioned and drawn with their terminus in 2006. Unpublished report from Patrick Gassmann (2007), Laboratoire de dendrochronologie, Office du Patrimoine et de l’Archéologie, Laténium, Espace Paul Vouga 7, CH-2068 Hauterive, Switzerland.
(TIF)
