# Supplementary figures and images for: Wild European Apple (Malus sylvestris (L.) Mill.) Population Dynamics: Insight from Genetics and Ecology in the Rhine Valley. Priorities for a Future Conservation Programme
Source: PLoS One. 2014 May 14;9(5):e96596. doi: 10.1371/journal.pone.0096596 (PMC4020776; doi:10.1371/journal.pone.0096596)

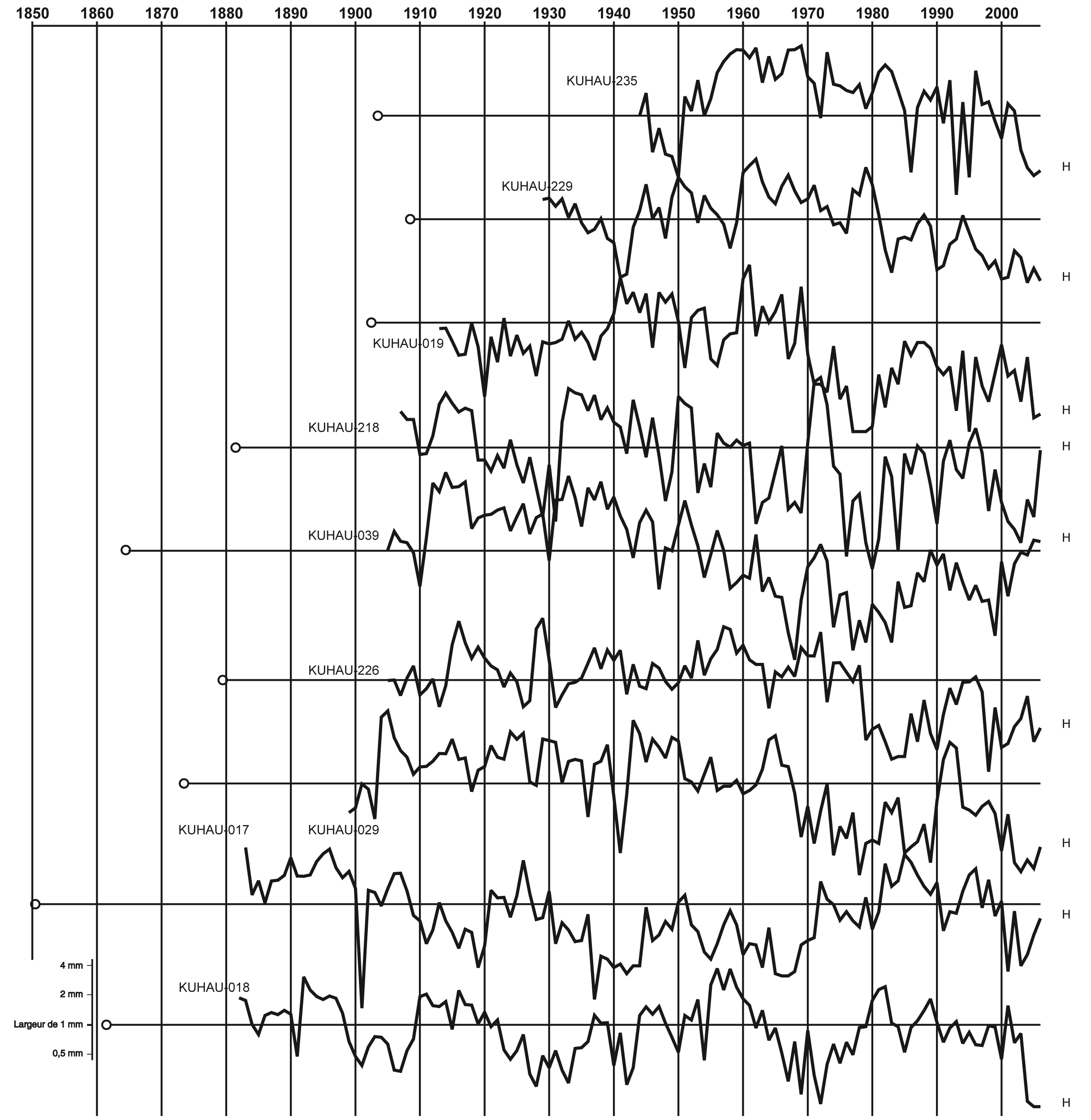

Supplement: Figure S1 — Diagram of dendrochronological sequences from wild apple trees in the Erstein forest (Kuhau site). The nine dendrochronological sequences shown in this diagram illustrate the growth characteristics of wild apples in the floodplain forest of Erstein. The horizontal line, which represents a growth ring with a width of 1 mm, provides a benchmark for assessing periods of growth and no growth. The circle drawn to the left of each sequence indicates the estimated pith location, as coring to the pith could not be done because of the hardness of the wood. The first five to ten years of difficult growth are generally followed by 70 years of vigorous growth and then a final 40 years or so of slower growth, as evidenced by very thin rings (example of KUHAU-029). The great variations in growth for each of the analysed wild apples made it impossible to establish correlations among their dendrochronological sequences. These sequences were thus positioned and drawn with their terminus in 2006. Unpublished report from Patrick Gassmann (2007), Laboratoire de dendrochronologie, Office du Patrimoine et de l’Archéologie, Laténium, Espace Paul Vouga 7, CH-2068 Hauterive, Switzerland. (TIF) [file pone.0096596.s001.tif]
